# The effects of body position on the distribution of obstructive, mixed and central sleep apnoea

**DOI:** 10.7196/AJTCCM.2019.v25i4.024

**Published:** 2019-12-06

**Authors:** G van der Colff, P R Bartel, P Becker, L T Hazelhurst

**Affiliations:** 1 Department of Biomedical Sciences, Faculty of Science, Tshwane University of Technology, Pretoria, South Africa; 2 Steve Biko Academic Hospital, Pretoria, South Africa; 3 Research Unit, Faculty of Health Sciences, University of Pretoria, South Africa

**Keywords:** sleep apnea, obstructive apnea, mixed apnea, central apnea, polysomnography

## Abstract

**Background:**

Obstructive sleep apnoea is commonly aggravated by the supine body position. The impact of body position on the severity of mixed and central sleep apnoeas is understudied.

**Objectives:**

To evaluate the impact of body position on obstructive, mixed and central apnoea indices in subjects presenting with this triform of sleep apnoea during a single polysomnogram.

**Methods:**

We retrospectively analysed 26 polysomnograms where obstructive, mixed and central apnoeas each occurred at a rate >5/hr. Comparisons between lateral and supine body positions were made for obstructive apnoea index (OAI), mixed apnoea index (MAI), central apnoea index (CAI), apnoea-hypopnoea index (AHI) and obstructive apnoea-hypopnoea index (OAHI).

**Results:**

Mean (SD) apnoea indices were significantly lower in lateral v. supine positions, respectively: MAI 15.06 (18.34) v. 32.09 (17.05); p<0.001, CAI 11.82 (11.77) v. 23.82 (14.18); p<0.001, AHI 79.46 (31.17) v. 99.47 (26.33); p<0.001, OAHI 67.87 (28.25) v. 76.00 (23.21); p=0.039. Unexpectedly, the converse was seen for OAI when comparing the lateral v. supine position: 53.10 (30.64) v. 43.58 (25.83); p=0.009, respectively.

**Conclusion:**

It may be beneficial for subjects with a combination of obstructive, mixed, and central apnoeas to avoid the supine body position. In this triform phenotype, mixed apnoeas are neither purely obstructive nor purely centrally mediated. Furthermore, obstructive, mixed, and central apnoeas may be different representations of a single respiratory abnormality.

## Background

Sleep apnoea is a breathing disorder characterised by recurrent, partial or complete pauses in respiration.^[1]^ Two major types of sleep apnoea syndromes are recognised, each with its own underlying pathophysiology. Obstructive sleep apnoea syndrome is largely the result of upper airway instability and collapse, while central sleep apnoea syndrome is more complex and is attributed to insufficient or absent central ventilatory drive,^[2]^ often secondary to cardiac or neurological disease.^[1]^ In overnight polysomnography, however, regarded as the preferred laboratory test for the evaluation of sleep-related breathing disorders,^[3]^ three major types of sleep apnoea are measured: obstructive, mixed and central apnoeas.^[1]^ The classification of mixed apnoeas remains unclear. The American Academy of Sleep Medicine (AASM) suggests that mixed apnoeas are part of obstructive apnoeas, despite displaying polysomnographic (PSG) features of both central and obstructive apnoeas. Furthermore, mixed apnoeas do not always respond well to continuous positive airway pressure therapy, the preferred treatment method of obstructive sleep apnoea syndrome.^[4]^ During the treatment of such cases, the apnoea indices of patients with predominantly mixed or obstructive apnoeas may either decrease in severity without achieving normalisation, or an unfavourable central apnoea response may be inadvertently induced.^[5]^ It is well established that body position during sleep has an impact on the severity of obstructive sleep apnoea, where the lateral position tends to be less severe than the supine position. The occurrence of obstructive apnoeas can be twice as frequent in the supine body position v. the lateral body position, an effect known as positional dependent obstructive sleep apnoea.^[6]^ With the exception of Cheyne-Stokes breathing, the impact of body position on other forms of central sleep apnoea is an area that is understudied.^[7]^

## Objectives

This study builds on previous sleep disorder studies by examining the impact of body position on the severity of obstructive, mixed and central sleep apnoeas. By not limiting our study participants to only cardiovascular-compromised patients, we were able to evaluate various other types of central sleep apnoea in addition to Cheyne-Stokes breathing. In addition, this study investigated whether mixed apnoeas, which display characteristics of both obstructive and central apnoeas, were strictly of an obstructive origin.

## Methods

### Study design

Subjects were eligible for this study if they were referred to the sleep laboratory (Pretoria, South Africa) as a standard referral for the evaluation of possible sleep-disordered breathing between 2009 and 2016. Retrospective data were collected after a search of the sleep laboratory’s database for polysomnograms matching our criteria. Verbal consent was obtained after a structured telephonic interview of subjects. Formal ethical approval was obtained from the Tshwane University of Technology Faculty of Science Committee for Research Ethics (ref. no. FCRE 2016/04/003).The primary PSG criteria for inclusion were an obstructive apnoea index (OAI), mixed apnoea index (MAI) and central apnoea index (CAI) of >5 per hour of sleep and occurring within a single overnight polysomnogram. Subjects >18 years of age were required to spend a minimum of 30 minutes in (i) each of the lateral positions and (ii) the supine body position. Subjects who were unable to change their body position at will (for example, during post-operative posturing or in the case of neuromuscular disorders) were excluded from the study.

### Polysomnography

Overnight polysomnography was performed either in a sleep laboratory or in a hospital ward. No home-based sleep studies were conducted. Subjects were not prompted to assume any specific body position during the polysomnogram recordings. All polysomnograms were acquired from dedicated PSG equipment (Alice PDx, Philips-Respironics Inc., USA) and were analysed using the device’s software (Alice Sleepware, version 2.8.78, Philips-Respironics Inc., USA).

PSG measurements included electroencephalography (C3 and C4 placements), electrooculography, submandibular electromyography, leg electromyography (anterior tibialis), electrocardiography, pulse rate and oxygen saturation via pulse oximetry, nasal airflow and snoring via nasal cannula, thoracic and abdominal respiratory effort via inductance plethysmography belt sensors, and body position via a built-in sensor within the recording device attached to the thorax at mid-sternal level.

PSG data were standardised by manual reanalysis in accordance with the criteria in the latest AASM scoring manual at the time of the study (version 2.2).^[8]^ Rule 1A for scoring hypopnoeas was used. No distinction was made between obstructive hypopnoeas and central hypopnoeas as per AASM guidelines.^[8]^ For practical reasons, oesophageal manometry was not performed during polysomnography and all hypopnoeas were assumed to be obstructive. Body positioning recording was derived from sensor readings that defined supine, left and right body positions. The lateral body position was recorded for left and/or right sensor readings. The OAI was defined as the sum of obstructive apnoeas and hypopnoeas. By this definition, the term obstructive apnoea when referring to a specific type of sleep apnoea includes hypopnoeas. The CAI was defined as the sum of central apnoeas and included Cheyne-Stokes breathing. The MAI was defined as the sum of all respiratory events that received a mixed apnoea scoring. The apnoea-hypopnoea index (AHI) was defined as the sum of all types of sleep apnoeas and detected in a polysomnogram.

The obstructive apnoea-hypopnoea index (OAHI) was defined as the index derived from the cumulative values of all obstructive apnoeas, hypopnoeas and mixed apnoeas as per AASM guidelines.^[8]^

### Statistical analysis

Measurements from each polysomnogram included the total sleep time, and sleep time in left, right, lateral, and supine body positions. The total number of apnoeas, apnoeas for each apnoea type and apnoeas in each body position was determined separately. Therefore, it was possible to determine the apnoea index of the left, right, lateral and supine body positions, for each of the OAI, CAI, MAI, OAHI,and AHI. These within-subject measurements were allocated to strata according to the apnoea index of each apnoea type respectively, and additionally, according to the apnoea index of the respective body positions. Statistical analysis was performed on Stata software (version 14, Stata Corp LLC,USA). In line with previous findings, ^[9,10]^ no significant differences in the apnoea indices between left and right body positions were found. Therefore, the left and right body position indices were combined, and defined the lateral body position index, which was henceforth used for comparing the severity of sleep apnoea, in terms of the apnoea index, of any given apnoea type, between the lateral and supine body position.

Inter-body position comparisons (lateral v. supine), of obstructive, central and mixed apnoeas, by means of the observation vector (OAI, CAI, MAI) was assessed using Hotelling’s paired *T²*-tests at a 0.05 level of significance, while specific differences between body positions were assessed using Student’s paired *t*-tests at a 0.0167 Bonferroni adjusted level of significance. Furthermore, inter-body position comparisons of the AHI and OAHI (which are linear functions of the OAI, CAI, and MAI), were respectively assessed using Student’s paired *t*-tests at a 0.05 level of significance. To compare obstructive, central, and mixed apnoeas by body position (intra-body position comparisons), random-effects Generalised Least Squares (GLS) regression was performed at a 0.05 level of significance, with the index type (OAI, CAI, and MAI) as fixed effect, and participant specified as the random component with an intercept. Linear predicted means, together with their 95% confidence intervals (CI) were reported by index over body position.

## Results

A total of 31 potential candidates (1.1%) were identified from a database of 2 802 patients, of whom 5 could not be reached for consent. Included in the study were 26 subjects between the ages of 28 and 85 years of whom 24 were male. The demographic data of these subjects are summarised in Table 1. Subjects were generally obese, with the body mass index (BMI) exceeding 30 kg/m² in 21 (81%) subjects. Cheyne-Stokes breathing was present in 2/26 subjects.

**Table 1:** Demographic data (N=26)

Variable	Mean (SD)	95% CI	Range
Age, years	50.22 (14.15)	44.50 - 55.94	28.90 - 85.20
BMI, kg/m²	34.41 (5.59)	32.15 - 36.67	26.70 - 49.40

SD = standard deviation

CI = confidence interval

BMI = body mass index

PSG analysis determined a mean (SD) total sleep time of 473.06 (50.97) minutes. Subjects had longer sleep times in the left v. right body position: mean (SD) 168.94 (125.21) v. 110.42 (86.59) minutes respectively and in the lateral v. supine body position: mean (SD) 279.36 (117.13) v. 178.00 (109.63) minutes, respectively. Four subjects completely avoided sleeping in the left position, while another 4 subjects avoided the right; however, they still met the inclusion criterion of >30 minutes of sleep in either one of the lateral positions. The AHI was severely abnormal (>30/hour) in all 26 subjects: mean (SD) index 88.16/hour (26.89). Mean (SD) indices for each body position for AHI were as follows: left 79.19/hour (29.77), right 78.27/ hour (32.60), lateral 79.46/hour (31.17) and supine 99.47/hour (26.33).

Inter-positional apnoea index comparisons showed that the CAI, MAI, AHI and OAHI were significantly lower in the lateral than the supine body position (Table 2). Unexpectedly, the OAI was significantly higher in the lateral compared to the supine body position. A Hotelling’s paired *T²*-tests with OAI + CAI + MAI as a single vector showed significant differences between lateral and supine body positions (p<0.001).

**Table 2:** Comparison of apnoea indices between lateral and supine body position (N=26)

Index Type	Body Position	Mean (SD)*	95% CI	p-value^†^
OAI	Lateral	53.10 (30.64)	40.72 - 65.47	0.009
	Supine	43.58 (25.83)	33.14 - 54.01	
CAI	Lateral	11.82 (11.77)	7.07 - 16.58	<0.001
	Supine	23.82 (14.18)	18.09 - 29.54	
MAI	Lateral	15.06 (18.34)	7.65 - 22.47	<0.001
	Supine	32.09 (17.05)	25.20 - 38.98	
AHI	Lateral	79.46 (31.17)	66.87 - 92.05	<0.001
	Supine	99.47 (26.33)	88.84 - 110.11	
OAHI	Lateral	67.87 (28.25)	56.46 - 79.28	0.039
	Supine	76.00 (23.21)	66.62 - 85.38	

SD = standard deviation

CI = confidence interval

OAI = obstructive apnoea index

CAI = central apnoea index

MAI = mixed apnoea index

AHI = apnoea-hypopnoea index

OAHI = obstructive apnoea-hypopnoea index

* Apnoea index units in events/hour.

^†^ Comparisons were made between body positions using a Student’s paired t-test with: (i) p<0.0167 considered significant after Bonferroni correction for OAI, CAI and MAI; (ii) p<0.05 considered significant for overall apnoea index (AHI or OAHI).

Random-effects GLS regression was used to compare the intrapositional distribution of obstructive, central and mixed apnoeas; thus, the OAI, CAI, and MAI were compared within the lateral and supine body positions, respectively (Table 3). While the CAI and MAI were comparable, both were significantly lower than the OAI irrespective of body position. Of additional interest was to investigate whether body position affected the severity of obstructive, central and mixed sleep apnoea during rapid eye movement (REM) sleep; however, only one subject m*et al*l the inclusion criteria during REM sleep and therefore this analysis was not performed.

**Table 3:** Intra-position comparison of the apnoea indices (events/hour) between apnoea types (N=26)

			p-value^†^	
Body Position	Index Type	Mean (95% CI)*	v. OAI	v. CAI
	OAI	53.10 (44.75 - 61.44)	-	-
Lateral	CAI	11.82 (3.48 - 20.17)	<0.001	-
	MAI	15.06 (6.71 - 23.40)	<0.001	0.591
	OAI	43.58 (36.02 - 51.13)	-	-
Supine	CAI	23.82 (16.26 - 31.37)	<0.001	-
	MAI	32.09 (24.53 - 39.64)	0.035	0.129

CI = confidence interval

OAI = obstructive apnoea index

CAI = central apnoea index

MAI = mixed apnoea index

* Apnoea index units in events/hour.

^†^ Random-effects Generalised Least Squares regression with p<0.05 considered significant.

## Discussion

The sample group in this study was representative of a severe obstructive sleep apnoea syndrome(mean AHI 88.16/hour), presenting mostly with obstructive apnoeas but also with a significant proportion of mixed and central apnoeas. This triform phenotype of sleep apnoea may be considered a rare phenotype, with a prevalence of less than 5%.^[4]^ The most important finding in this study was that while all apnoea indices were above the normal threshold in both the supine and lateral body positions, 4/5 apnoea indices (CAI, MAI, OAHI and AHI) were significantly lower in the lateral body position. The exception was the OAI, where the severity of obstructive apnoeas was significantly higher in the lateral compared to the supine body position. While it is well established that obstructive sleep apnoea is more severe in the supine position,^[11]^ this was evident in less than a third (27%) of the subjects in this study. For the OAHI, however, which represented all obstructive-mediated apnoeas collectively, sleep severity relative to body position was consistent with findings from other studies.^[12]^ Considering that the OAHI, MAI and OAI are calculated from obstructive-mediated events, it is unclear why the OAI deviates from the MAI and the OAHI findings with respect to severity in supine v. lateral body positions. Three possible explanations are proposed: (*i*) in subjects who present with a combination of obstructive, mixed and central apnoeas, reflex inhibition of respiratory effort, mediated via receptors in the mucosa of the upper airway, may be triggered upon airway collapse associated with obstructive apnoeas, specifically while in the supine position. This may result in an increase in the occurrence of central and mixed apnoeas during supine sleep, as upper airway collapse is known to be aggravated by this body position;^[13]^ (*ii*) subjects presenting with this triform of sleep apnoea evaluated in the current study, have been associated with altered, or increased respiratory control instability, that may have contributed to the atypical distribution of obstructive apnoeas;^[4,14]^ (*iii*) obstructive apnoea indices vary in the lateral position whereas are more stable in the supine position.^[15]^ The OAHI, which represents all obstructive-mediated apnoeas ^[8]^ was significantly lower in the lateral compared to the supine body position as expected. Therefore, it could be argued that mixed apnoeas contributed to the obstructive-mediated apnoea index severity in relation to body position. Furthermore, mixed apnoeas displayed characteristics of central apnoeas with respect to severity and body position. Therefore, based on patterns in severity between supine and lateral sleeping positions, the MAI displayed both obstructive and centrally mediated sleep apnoea characteristics. Given the similarities between the OAHI, CAI and MAI with respect to severity and body position, it is feasible that obstructive, mixed and central apnoeas are of common origin, manifesting as this triform phenotype of sleep apnoea. This hypothesis is in keeping with that of Issa and Sullivan,^[13]^ who suggested that in a specific subgroup of sleep apnoea, where subjects presented with a combination of obstructive, mixed and central apnoeas, the occurrence of different types of sleep apnoea (that appear to be body position dependent) are probably different representations of a single respiratory abnormality. While this study was not restricted to heart-failure patients and those with Cheyne-Stokes respiration occurred only in a minority of subjects, both the MAI and CAI were significantly lower in the lateral v. supine body position, respectively, which supports previous findings.^[9,16]^ Szollosi *et al*. ^[9]^ also showed that in heart-failure patients, the CAI (central apnoeas associated with Cheyne-Stokes breathing) was more than 50% less severe in the lateral compared to the supine body position. We noted that in participants presenting with the triform phenotype of sleep apnoea, a change in body position (from supine to lateral) resulted in a reduction of ~50% for the CAI and ~47% for the MAI.

Other studies have noted that those suffering from Cheyne-Stokes breathing and congestive heart failure prefer sleeping in the right body position, ^[9,10,16]^ however only two subjects in this study presented with Cheyne-Stokes breathing, therefore the finding that most subjects preferred the left sleeping position was interesting. Subjects spent more sleep time in the lateral body position as well, supported by reports by Szollosi *et al*. ^[9]^ In contrast, Eiseman *et al*. ^[17]^ found that more than half of the sleep time recorded within a sleep laboratory environment is expected to be in the supine body position. This may be a result of major differences in subject selection between studies. Both Szollosi *et al*. ^[9]^ and the present study included subjects where central apnoeas were prominent PSG features (median 124 events/ hr), whereas Eiseman *et al*. ^[17]^ specifically studied obstructive apnoeas and central apnoeas were negligible in their study (median 2 events/ hr). This raises the question of whether subjects with PSG features that include a significant number of central apnoeas, compensate for their disturbed breathing by favouring a lateral body position. Further studies in this regard are warranted.

## Conclusion

The lateral sleeping position is associated with a decrease in the severity of obstructive-mediated (OAHI) and centrally mediated (CAI) apnoeas in subjects who present with a combination of obstructive, mixed and central sleep apnoeas. Furthermore, we propose that in this specific sleep apnoea phenotype, mixed apnoeas are neither purely obstructive nor purely central-mediated in origin. Obstructive, mixed and central sleep apnoea may be different representations of a single respiratory abnormality in patients presenting with this triform of sleep apnoea. Further studies on the effectiveness of positional therapy as a potential treatment option in this phenotype may be of value.
